# Dietary Glycine Prevents FOLFOX Chemotherapy-Induced Heart Injury: A Colorectal Cancer Liver Metastasis Treatment Model in Rats

**DOI:** 10.3390/nu12092634

**Published:** 2020-08-28

**Authors:** Juste Maneikyte, Augustinas Bausys, Bettina Leber, Nicole Feldbacher, Gerald Hoefler, Dagmar Kolb-Lenz, Kestutis Strupas, Philipp Stiegler, Peter Schemmer

**Affiliations:** 1 General, Visceral and Transplant Surgery, Department of Surgery, Medical University of Graz, 8036 Graz, Austria; juste.maneikyte@gmail.com (J.M.); abpelikanas@gmail.com (A.B.); bettina.leber@medunigraz.at (B.L.); Nicole.Feldbacher@medunigraz.at (N.F.); philipp.stiegler@medunigraz.at (P.S.); 2 Faculty of Medicine, Vilnius University, 03101 Vilnius, Lithuania; kestutis.strupas@santa.lt; 3 National Cancer Institute, 08406 Vilnius, Lithuania; 4 Diagnostic and Research Institute of Pathology, Medical University of Graz, 8010 Graz, Austria; gerald.hoefler@medunigraz.at; 5 Institute of Cell Biology, Histology and Embryology, Medical University Graz, 8010 Graz, Austria; dagmar.kolb-lenz@medunigraz.at; 6 Center for Medical Research, Core Facility Ultrastructure Analysis, Medical University Graz, 8010 Graz, Austria

**Keywords:** glycine, FOLFOX, cardiotoxicity, colorectal cancer

## Abstract

Introduction: FOLFOX chemotherapy (CTx) is used for the treatment of colorectal liver metastasis (CRLM). Side effects include rare cardiotoxicity, which may limit the application of FOLFOX. Currently, there is no effective strategy to prevent FOLFOX-induced cardiotoxicity. Glycine has been shown to protect livers from CTx-induced injury and oxidative stress, and it reduces platelet aggregation and improves microperfusion. This study tested the hypothesis of glycine being cardioprotective in a rat model of FOLFOX in combination with CRLM. Materials and Methods: The effect of glycine was tested in vitro on human cardiac myocytes (HCMs). To test glycine in vivo Wag/Rij rats with induced CRLM were treated with FOLFOX ±5% dietary glycine. Left ventricle ejection fraction (LVEF), myocardial fibrosis, and apoptosis, also heart fatty acid binding protein (h-FABP) and brain natriuretic peptide levels were monitored. PCR analysis for Collagen type I, II, and brain natriuretic peptide (BNP) in the heart muscle was performed. Results: In vitro glycine had no effect on HCM cell viability. Treatment with FOLFOX resulted in a significant increase of h-FABP levels, increased myocardial fibrosis, and apoptosis as well as increased expression of type I Collagen. Furthermore, FOLFOX caused a decrease of LVEF by 10% (*p* = 0.028). Dietary glycine prevented FOLFOX-induced myocardial injury by preserving the LVEF and reducing the levels of fibrosis (*p* = 0.012) and apoptosis (*p* = 0.015) in vivo. Conclusions: Data presented here demonstrate for the first time that dietary glycine protects the heart against FOLFOX-induced injury during treatment for CRLM.

## 1. Introduction

Colorectal cancer (CRC) is the third most common cancer worldwide [1]. More than half of those patients develop liver metastasis requiring chemotherapy (CTx) in the neoadjuvant, adjuvant, or palliative setting [2,3]. FOLFOX, which consists of 5-fluorouracil (5-FU), leucovorin, and oxaliplatin (OX) is proven to be effective for colorectal cancer liver metastasis (CRLM) treatment [4]. However, the use of these cytotoxic agents is associated with numerous side effects including cardiotoxicity, which is a severe and even potentially lethal complication [5]. The reported incidence of 5-FU-induced cardiotoxicity varies between 1% and 68% for various solid cancers [6,7,8], with a rate of 8.5% in a specific colorectal cancer patients cohort [9]. The exact pathophysiologic mechanisms for FOLFOX-induced cardiotoxicity are not fully clarified but both agents—5-FU and OX—are known to be cardiotoxic, and several mechanisms including coronary thrombosis, arteritis, and vasospasm have been proposed [10]. Some studies show direct 5-FU-mediated cytotoxic effects on the endothelium resulting in the release of vasoactive substances and development of a prothrombogenic status [11] or direct toxic effects of 5-FU on cardiomyocytes, interaction with the coagulation system, and autoimmune responses [10]. The exact mechanisms for platinum-based drugs’ cardiotoxicity remains unknown as well [12]. Currently, few hypotheses exist including the secondary toxic effect due to nephrotoxicity, the potential negative effect on the sinoatrial node, or direct damage by reactive oxygen species [12,13]. Additionally, the incidence of cardiotoxicity seems to increase when 5-FU and OX are combined [14,15]. As FOLFOX-induced cardiotoxicity can cause irreversible damage to the myocardium, preventive strategies are mandatory.

Glycine is a simple amino acid with known biological activity. Experimental studies show that glycine reduces human and rat platelet aggregation in vitro [16], improves microcirculation [17], has antioxidative effects [18,19] as well as anti-inflammatory, immunomodulatory, and direct cytoprotective properties [20]. Moreover, glycine was shown to have anti-tumorigenic properties in a previously described animal model of CRLM induced by CC531 cells [21].

Therefore, we hypothesized, that glycine may work as a cardioprotective substance against FOLFOX-induced cardiotoxicity in vitro and in vivo. This study aimed to demonstrate the cardioprotective potential of glycine on rats suffering from CRLM and treated with a single cycle of FOLFOX CTx.

## 2. Materials and Methods 

### 2.1. Cell Culture

#### 2.1.1. Cell Line

Human cardiac myocytes (HCMs, Promo Cell, Heidelberg, Germany) were cultivated at humified atmosphere at 37 °C, 5% CO_2_ using ready-to-use Myocyte Growth Medium (Promo Cell, Heidelberg, Germany). The culture medium was renewed every second or third day, and cells were re-seeded whenever 90% confluence was reached.

A rat colorectal cancer cell line (CC531) (Cell Lines Service, Eppelheim, Germany) was cultivated at 37 °C, 5% CO_2_ using RPMI-1640 medium supplemented with 10% fetal bovine serum (GE Healthcare Life Sciences, Logan, UT, USA), 1% penicillin/streptomycin, 1% L-glutamine, and 25 mM HEPES as described previously [21]. The culture medium was renewed every second or third day, and cells were re-seeded whenever 90% confluence was reached.

#### 2.1.2. Cell Proliferation and Viability Assay

To determine the effect of glycine on HCMs, 1.5 × 10^4^ cells per well were seeded in 96-well plates and grown for 24 hours at standard conditions in glycine-free media. Subsequently, conditioning by supplementation of media with various concentrations of glycine (Control (0), 0.05, 0.1, 0.25, 0.5, 1, 2, 4, and 8 mmol/L; Carl Roth, Karlsruhe, Germany) for 48 hours was done. After incubation, cell viability was measured by the 3-(4,5-dimethylthiazol-2-yl)5-diphenyltetrazolium bromide (MTT; Sigma Aldrich, St. Louis, MO, USA) assay according to the manufacturer’s guidelines. Briefly, MTT (Sigma Aldrich, St. Louis, MO, USA) solution was added to the cells and incubated for 2 hours at 37 °C. After discarding the supernatant, the precipitated formazan crystals were dissolved in dimethyl sulfoxide (Sigma Aldrich, St. Louis, MO, USA), and the optical density was measured after 30 minutes at 570 nm by a SPECTROstar microplate reader (BMG Labtech, Ortenberg, Germany). Cell viability was calculated as the percentage of vehicle control. All experiments were performed in duplicates and repeated three times.

### 2.2. Animal Experiments

All animal experiments were performed in accordance with 3Rs, principles of laboratory animal care, and the Austrian national laws. Republic of Austria federal ministry of education, science, and research approval (BMWFW-66.010/0130-WF/V/3b/2017) was obtained before this study was conducted. Seven-week-old male Wag/Rij rats (weight 150–220 g) were purchased from Charles River (Wilmington, MA, USA) and kept in the animal facility of the Medical University of Graz under standard laboratory conditions. A 12:12 h light:dark cycle was maintained. All the animals had access to water and food ad libitum. Weighing and blood drawing were done on the first experimental day and days 6, 14, 17, and 21. All study-related procedures were performed at approximately the same daytime to avoid bias caused by natural circadian rhythms.

### 2.3. Experimental Protocol 

**Figure 1:**
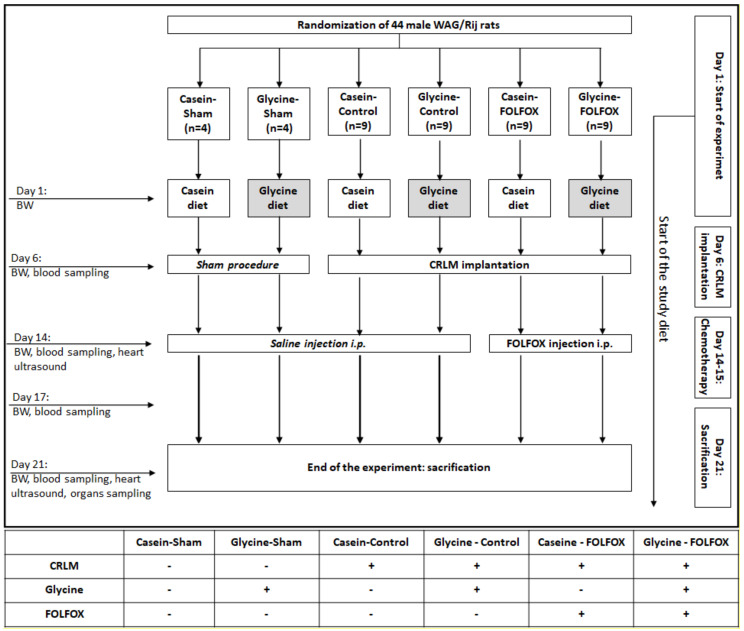
Flowchart of the animal experiment. FOLFOX—chemotherapy regimen containing calcium folinate, oxaliplatin, and 5-fluorouracil; BW—body weight; CRLM—colorectal cancer liver metastasis; i.p. —intraperitoneally.

The study design is shown in Figure 1. Forty-four animals were randomized into 6 groups. On the first day of the study, regular rat chow was replaced by study diets. Half of the animals (*n* = 22) received a 5% glycine-enriched diet (glycine diet: modified C1000 diet 15% casein plus 5% glycine; Altromin Spezialfutter, Lage, Germany). The other animals (*n* = 22) received a 20% casein diet for isonitrogenous balance as a control diet (control diet: 20% casein; Altromin Spezialfutter, Lage, Germany) since casein is a standard control for dietary glycine as described previously [22]. After 6 days, CRC liver metastasis was induced by injecting CC531 cells into the right liver lobe as described below. Sham animals (*n* = 8) underwent the same procedures, with an injection of cell-free PBS. Eight days after tumor implantation, a heart ultrasound to evaluate heart function was performed as described below. Immediately after imaging, animals received one cycle of FOLFOX CTx, which consisted of 200 mg/m^2^ calcium folinate (Sandoz, Holzkirchen, Germany), 85 mg/m^2^ OX (Fresenius Kabi, Bad Homburg, Germany), and 1000 mg/m^2^ 5-FU (Sandoz, Holzkirchen, Germany) injected intraperitoneal. Twenty-four hours after the first dose, the second dose of 200 mg/m^2^ calcium folinate and 1000 mg/m^2^ 5-FU was applied in the same way. The doses were calculated according to the animals’ skin surface as described elsewhere [22]. Control animals received an equivalent volume of saline. Seven days after the FOLFOX application, the heart ultrasound was repeated immediately before animals were sacrificed for organ and blood collection.

### 2.4. Tumor Implantation

For tumor implantation, anesthesia was induced by isoflurane (2%, 2 L/min) and fentanyl (5 µg/kg, i.m.) application. Animals were placed in a supine position on an automatically regulated heating pad to maintain a body temperature of 37 °C during the procedure. Under continuous isoflurane (2%, 2 L/min) inhalation, a right subcostal incision was performed. The right liver lobe was mobilized, and 100 µL (5 × 10^6^ cells) of cell suspension in PBS was injected under the liver capsule. The laparotomy was closed by two layers of interrupted stitches (Vicryl 4-0, Ethicon, Somerville, NJ, USA). The skin was adapted and glued with tissue glue (Mayer-Haake, Ober-Mörlen, Germany). The procedure-related pain was managed with a single subcutaneous injection of carprofen (4 mg/kg) and by addition of ibuprofen (0.4 mg/mL) to the drinking water for 4–5 days.

### 2.5. Heart Ultrasound

Inhalational anesthesia was induced with isoflurane (2%, 2 L/min) and maintained during the procedure. Heart ultrasound was performed using the VisualSonics Vevo 770® High-Resolution Imaging System (VisualSonics Inc., Toronto, Canada). Left parasternal short-axis views of the left ventricle (LV) at the level of papillary muscles were used to define the internal diameters of the LV. Left ventricular end-diastolic diameter (LVEDD) and left ventricular end-systolic diameter (LVESD) were determined from the obtained M-mode pictures. The ejection fraction was calculated by the Teichholz formula as described elsewhere [23].

### 2.6. Blood Sample Analysis

Venous blood samples were collected from the subclavian vein under isoflurane anesthesia. Complete blood count (CBC) was measured using V-Sight hematology analyzer (A. Menarini Pharma GmbH, Vienna, Austria).

A commercially available enzyme-linked immunosorbent assay (ELISA) kit was used to measure concentrations of heart fatty acid-binding protein (h-FABP) (Cusabio Biotech, Newark, NJ, USA) and brain natriuretic peptide (BNP) (Abcam, Cambridge, United Kingdom) in plasma samples collected on the last experimental day. Tests were performed according to the manufacturer’s instruction.

### 2.7. Histology and Immunohistochemistry

Collagen in hearts was stained using chromotrope aniline blue (CAB) staining in a routine pathology lab in Medical University of Graz on sections from formalin-fixed, paraffin-embedded (FFPE) specimens. Stained slides were scanned using the Apperio AT (Wetzlar, Germany) slide scanner and viewed using the Aperio ImageScope ver.12.3.2.8013 software (Leica Biosystems, Wetzlar, Germany). Quantification of CAB staining was performed in 5 randomly selected areas in each heart. Snapshots at 100× magnification were taken and analyzed using the ImageJ software (U.S. National Institutes of Health, Bethesda, MD, USA), giving a quantification based on the image color analysis. The fibrosis index was calculated as percentage of blue color per picture.

For immunohistochemistry, 3 µm sections of FFPE heart samples were used. To evaluate apoptosis anti-caspase 3 antibody (Abcam, Cambridge, UK; dilution 1:200, rabbit polyclonal) was used in combination with the UltraVision LP Detection System HRP Polymer (Thermo Fisher Scientific, Waltham, MA, USA) and DAB chromogen (Dako, Via Real Carpinteria, CA, USA). For positive control, rat thymus tissue was stained. For negative control, primary antibodies were omitted. Stained slides were scanned and viewed using the same optical system and software as mentioned above.

Quantification of Caspase 3 was performed in 5 randomly selected heart areas from each slide. Snapshots at 100× magnification were taken and analyzed using ImageJ software. For Caspase 3 analysis, IHC and Classic Watershed plugins were used. The apoptotic index was calculated as the ratio of positively stained nuclei to the total number of nuclei per view.

### 2.8. Electron Microscopy

For electron microscopy, heart tissue was fixed in 2.5% (wt/vol) glutaraldehyde and 2% (wt/vol) paraformaldehyde in 0.1 mol/L phosphate buffer, pH 7.4, for 2 h, postfixed in 1% (wt/vol) osmium tetroxide for 2 h at room temperature. After dehydration (graded series of ethanol), tissue was infiltrated (ethanol and agar 100 epoxy resin, pure agar 100 epoxy resin) and placed in agar 100 epoxy resin (8 h), transferred into embedding molds, and polymerized (48 h, 60 °C).

Ultrathin sections (70 nm) were prepared by a UC 7 Ultramicrotome (Leica Microsystems, Vienna, Austria) and stained with lead citrate for 5 min and platin blue for 15 min. Images were taken using a Tecnai G2 20 transmission electron microscope (FEI, Eindhoven, Netherlands) with a Gatan UltraScan 1000 charge-coupled device (CCD) camera at −20 °C (acquisition software Digital Micrograph; Gatan, Munich, Germany). The acceleration voltage was 120 kV.

### 2.9. Quantitative Polymerase Chain Reaction

#### 2.9.1. RNA Isolation and Reverse Transcription

Heart tissue samples were snap-frozen and stored in liquid nitrogen until nucleic acid extraction. Then, 50–100 mg of tissue was homogenized in 1 mL TRIzol reagent in combination with a MagNA Lyser (Roche Diagnostics GmbH, Mannheim, Germany). Isolation of RNA was done according to the protocol provided by the manufacturer. Quality and quantity were determined by Nanodrop 2000 (Thermo Fisher Scientific, Waltham, MA, USA). Two micrograms of RNA was used for reverse transcription (High-Capacity cDNA RT Kit; Thermo Fisher Scientific, Waltham, MA, USA) according to the protocol provided by the manufacturer in a final volume of 20 µL.

#### 2.9.2. Real-Time PCR

Real-time PCR amplification and melting analysis were performed based on an already published method [24] using a BioRad CFX96 Touch^TM^ System (Bio-Rad Laboratories Ges.m.b.H., Vienna, Austria). cDNA corresponding to an equivalent of 5 ng RNA was added to a reaction mix containing Promega GoTaq® qPCR Master Mix (Promega, Madison WI, USA) containing 1 µM of each primer in a final reaction volume of 10 μL. The PCR reaction mixture was subjected to an initial denaturation at 95 °C for 10 seconds, followed by 45 cycles of denaturation at 95 °C for 10 seconds, annealing at 58 °C at 20 seconds and elongation at 72 °C for 30 seconds followed by a melting curve (60 to 95 °C). For detailed information of primers used see Table 1.

Gene expression was determined using the Bio-Rad CFX Manager 3.1 (Bio-Rad Laboratories Ges.m.b.H., Vienna, Austria) using the Cq regression method embedded in the program. All PCR reactions were done in duplicates. Data were normalized to the housekeeping gene (*ACTB*) and fold change was calculated as the ratio of the target gene expression in the experimental groups.

### 2.10. Statistical Analysis

Statistical analysis was performed using SPSS v.20.0 (SPSS Inc., Chicago, IL, USA). Data are presented as median and quartiles (Q1, Q3) unless stated differently. Differences between groups were analyzed using non-parametric tests, i.e., Mann–Whitney U test or Kruskal–Wallis test. Two-tailed Spearman test was used for continuous data correlation. All statistical tests were wo-sided. *p*-Values < 0.05 were considered statistically significant.

### 2.11. Ethics Approval and Consent to Participate

All animal experiments were performed in accordance with the principles of laboratory animal care and national laws. The study protocol was approved by the Austrian ministry for science, research, and economy (No. BMWFW-66.010/0130-WF/V/3b/2017; August 2017).

## 3. Results

### 3.1. Cell Experiments

**Figure 2:**
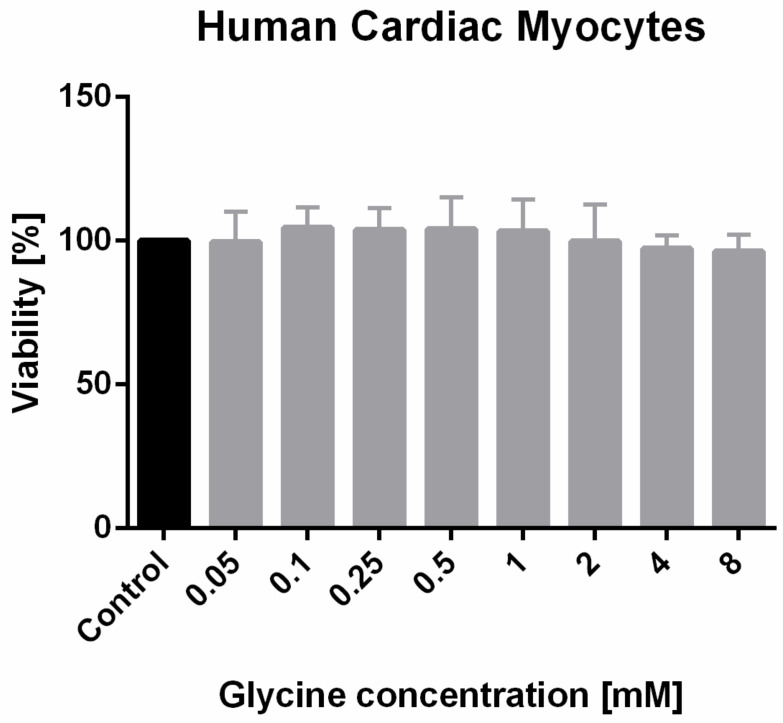
Glycine effect on HCM cell growth and proliferation by MTT (3-(4,5-dimethylthiazol-2-yl)5-diphenyltetrazolium bromide) assay. HCM cells viability after incubation with various concentrations of glycine measured by MTT assay and presented as the percentage of the controls.

Various concentrations of glycine did not have any significant impact on the viability of HCM cells (Figure 2).

### 3.2. Animal Experiments

#### 3.2.1. General Health Conditions and Body Weight 

Forty-three (97.7%) animals survived the whole experimental protocol. Animals were not affected by study-related procedures, other than FOLFOX, which resulted in diarrhea and weight loss. The median weight of animals on day 21 in FOLFOX groups with glycine or casein was 90% and 93% of baseline weight, respectively. In comparison, groups without FOLFOX weighted 123–129% of baseline weight, *p* < 0.05.

#### 3.2.2. Complete Blood Count

Hemoglobin (HGB) and red blood cell (RBC) levels remained stable throughout the whole study period in all study groups. FOLFOX induced a significant decrease in white blood cells (WBCs), which progressed until severe leukopenia (Table 2). Glycine did not affect HGB, RBC, or WBC levels. 

#### 3.2.3. Glycine Concentration in Serum

Animals treated with glycine diet had almost fivefold higher plasma glycine concentration compared to animals who received casein diet (955 (400; 1250) µmol/L vs. 190 (126; 285) µmol/L, *p* = 0.001).

#### 3.2.4. BNP and h-FABP Concentrations in Serum

**Figure 3:**
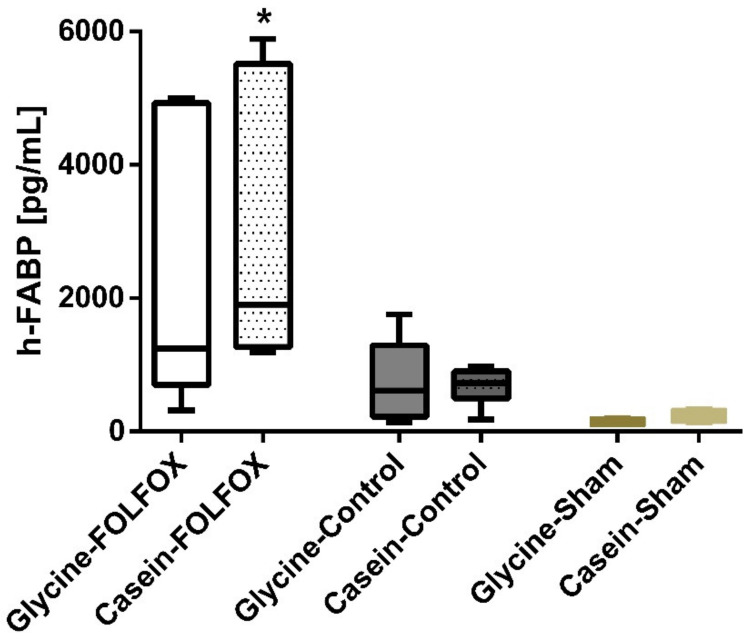
Serum levels of heart fatty acid binding protein (h-FABP) on the last experimental day. h-FABP level measured on the last experimental day was different between the study groups. * *p* < 0.05. The Casein–FOLFOX group had a significantly higher h-FABP level compared to groups that had not received chemotherapy (Glycine–Control; Casein–Control; Glycine–Sham; Casein–Sham). Although, the difference between Casein–FOLFOX and Glycine–FOLFOX groups was not significant.

BNP levels were not different between the study groups. FOLFOX significantly increased the h-FABP level to 1904 (1265; 5514) pg/mL compared to 728 (499; 900) pg/mL in corresponding control (*p* = 0.01) (Figure 3). Glycine reduced the increase of h-FABP after FOLFOX by 35%, however, the difference failed to be significant.

#### 3.2.5. Heart Ultrasound 

**Figure 4:**
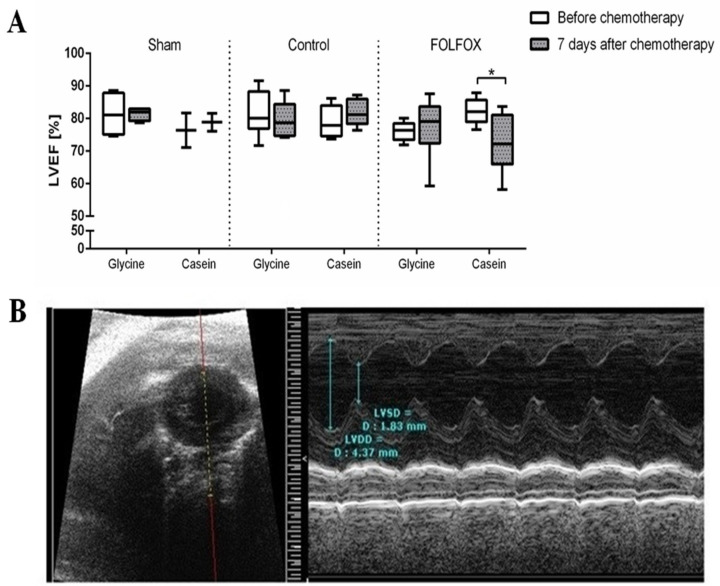
Left ventricle ejection fraction (LVEF) before and after chemotherapy in different diet groups. (**A**) Left ventricle ejection fraction (LVEF) before and after chemotherapy in the study groups; * *p* < 0.05. (**B**) Representative view from heart ultrasound. Left: Heart in parasternal short-axis view; Right: M-mode, left ventricular internal diameters.

Baseline values of LVEF measured before FOLFOX in different groups varied between 76% and 82%, *p* = 0.207. After FOLFOX application, LVEF significantly decreased from 82% (78; 85) to 72% (65; 81), *p* = 0.028 in the group treated with casein diet. In contrast, glycine prevented a decrease in LVEF after FOLFOX (Figure 4).

CRLM alone had no effect on LVEF (Figure 4). The decrease of LVEF in groups treated with FOLFOX significantly correlated with the serum h-FABP level (*R* = 0.572, *p* = 0.033) and fibrosis index in the myocardium (*R* = 0.731, *p* = 0.005). Although, apoptotic index in the myocardium, as well as BNP, type I and II collagen gene expression levels, showed no significant correlation with the decrease of LVEF.

#### 3.2.6. Fibrosis Index in the Myocardium

**Figure 5:**
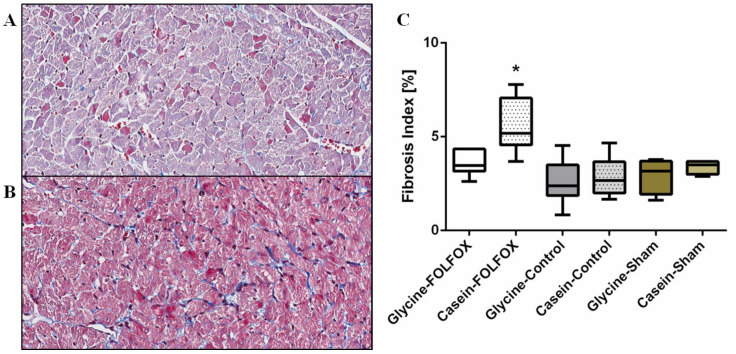
Fibrosis index measured by chromotrope aniline blue staining. Representative pictures at 100× magnification of chromotrope aniline blue stain for fibrous tissue in the myocardium of animals treated with FOLFOX and glycine (**A**) and FOLFOX alone (**B**). The amount of fibrosis between the study groups was different (**C**). * The fibrosis index in the group treated with FOLFOX alone was significantly higher compared to that of all other study groups, *p* < 0.05.

Figure 5 shows the fibrosis index in each study group measured by CAB staining. The highest myocardial fibrosis index of 5.1% (4.5; 7.0) was in the group treated with Casein–FOLFOX. Glycine prevented increased fibrosis following FOLFOX, while the fibrosis index was reduced by 33%, *p* = 0.012. Moreover, the fibrosis index in the Glycine–FOLFOX group was not significantly different from that of the groups without CTx.

#### 3.2.7. Apoptotic Index in the Myocardium

**Figure 6:**
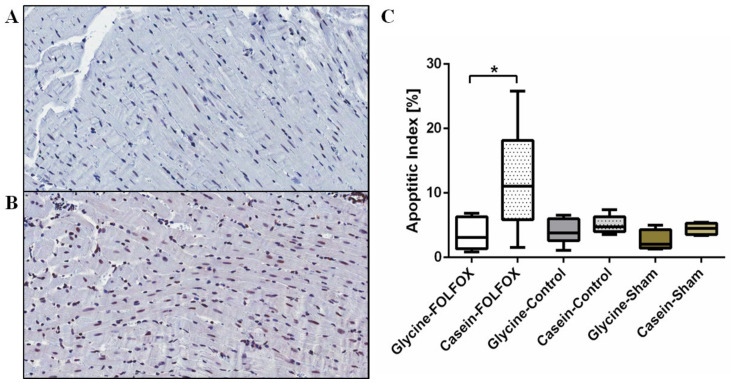
The apoptotic index measured by immunohistochemistry with anti-Caspase 3 antibody. Representative pictures at 100× magnification of anti-Caspase 3 staining for fibrous tissue in the myocardium of animals treated with FOLFOX and glycine (**A**) and FOLFOX alone (**B**). The apoptotic cell rate was significantly higher in the FOLFOX-alone group compared to that in the group treated with FOLFOX with glycine, * *p* < 0.05 (**C**).

FOLFOX induced the increase of apoptosis in the myocardium (Figure 6). Glycine prevented the FOLFOX-induced increase of apoptosis, while the apoptotic index was reduced by 3.5-fold, *p* = 0.015.

#### 3.2.8. Electron Microscopy of Heart Tissue

**Figure 7:**
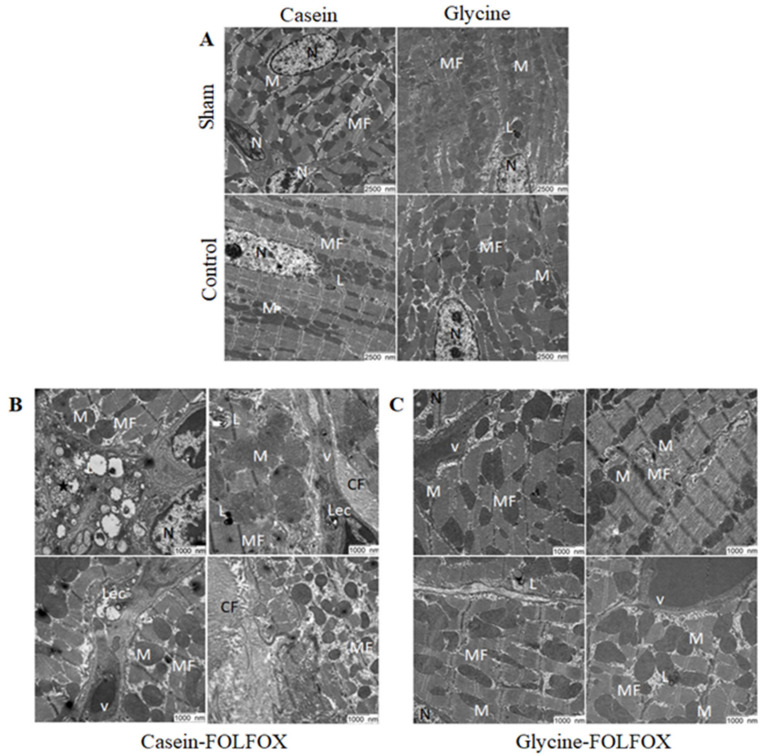
Transmission electron microscopy scans of heart samples. Representative pictures of hearts scanned by electron microscopy in (**A**) groups without FOLFOX (Casein–Sham, Glycine–Sham, Casein–Control, Glycine–Control) at magnification 7000×; (**B**) Casein–FOLFOX; and (**C**) Glycine–FOLFOX groups at magnification 12,000×. FOLFOX induced a significant increase of the collagen fibers in the heart muscle, and lysosomes within the endothelial cells were observed in the Casein–FOLFOX group. Glycine prevented FOLFOX-induced ultrastructural changes. MF—myofibrils; M—mitochondria; N—nucleus, L—lysosomes; Lec—lysosomes within endothelial cells; asterisk—lysosomes containing dark granules and other lipids; v—vessels; CF—collagen fibers.

Representative transmission electron microscopy images are shown in Figure 7. The groups without FOLFOX (Casein–Sham, Glycine–Sham, Casein–Control, and Glycine–Control) all showed normal architecture of the heart (Figure 7A). In the Casein–FOLFOX group, collagen fibers within the heart muscle and lysosomes within the endothelial cells were observed (Figure 7B), whereas those changes were absent in the Glycine–FOLFOX group (Figure 7C).

#### 3.2.9. qPCR of Heart Tissue

**Figure 8:**
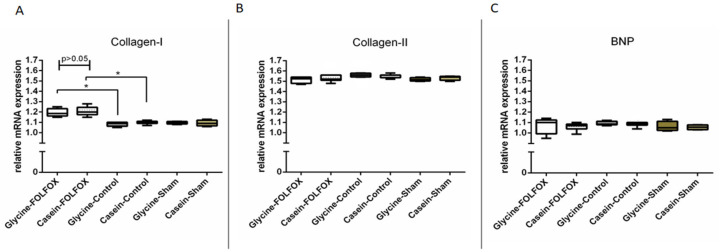
Type I and II collagen and brain natriuretic peptide (BNP) gene expression in the heart muscle by qPCR. Type I collagen gene expression was significantly increased in FOLFOX groups (Casein–FOLFOX; Glycine–FOLFOX) compared to that in corresponding controls (Casein–Control; Glycine–Control), * *p* < 0.05. Although, there was no difference between Casein–FOLFOX and Glycine–FOLFOX groups (**A**). Type II collagen (**B**) and BNP (**C**) expression was not different between the study groups.

The expression of type I collagen was upregulated in the FOLFOX groups compared to that in corresponding controls (*p* < 0.05). Although, there was no differences between Casein–FOLFOX and Glycine–FOLFOX groups (Figure 8A). Type II collagen (Figure 8B) and BNP (Figure 8C) gene expression was not different between the study groups.

## 4. Discussion

This study investigated the potential cardioprotective effect of the non-toxic amino acid, glycine, in a CRLM FOLFOX CTx rat model. The results demonstrate that glycine is involved in processes that have a significant cardioprotective effect by reducing the level of FOLFOX-induced fibrosis and apoptosis in the myocardial tissue and preserving LVEF.

The pyrimidine antimetabolite (5-FU) or its orally administered prodrug, capecitabine, is widely used in the treatment of breast, bladder, prostate, and various gastrointestinal cancers, including CRC [25]. However, the use and effectiveness are limited by cardiotoxicity, which most commonly occurs already after the first cycle of administration [26]. Moreover, the incidence of cardiotoxicity seems to be even higher when fluoropyrimidines are used in combination with OX [14,15] as used in the FOLFOX CTx scheme and in the presented experimental model. The pathogenesis of 5-FU-induced cardiotoxicity is still not fully clarified [27,28], and there are even less data on OX-induced cardiotoxicity [12]. The existing theories for 5-FU cardiotoxicity include vasoconstriction, endothelial injury leading to a procoagulant state, and direct myocardial cell toxicity [28,29,30,31]. Several previous preclinical studies suggested that fluoropyrimidines may induce direct cardiomyocyte injury via oxidative stress, the release of free radicals, and induction of nuclear alteration, cytoplasmic vacuolization, membrane injury, and autophagy [32,33,34]. All these changes finally trigger cell apoptosis and result in cardiotoxicity [28]. Moreover, the platinum-based agents may also cause direct, reactive oxygen species-mediated injury of the heart [12]. In this study, which supports the direct cardiomyocyte toxicity by FOLFOX, a more than threefold higher rate of apoptotic cells, increased expression of *type I collagen* gene in heart tissue, and myocardial fibrosis were detected after FOLFOX application. Besides the FOLFOX-induced pathologic changes in the myocardial tissue, impaired LV function was detected and the decrease of LVEF after FOLFOX correlated to h-FABP level and myocardial fibrosis. These results are in accordance with several recent preclinical studies, which also showed an increased level of apoptosis, myocardial fibrosis, and decreased LVEF after treatment with anthracycline-based chemotherapy [35,36,37,38] and with the series of reports that showed reduced LVEF following 5-FU-based chemotherapy in humans [39,40,41,42,43]. 

Interestingly, the myocardial injury marker, h-FABP level, was markedly elevated after FOLFOX CTx irrespective of diet but was noticeably lower in animals who received glycine. On the one hand, ElGhandour et al. [44] studied h-FABP in 40 patients treated with 6 cycles of anthracycline-based CTx and concluded that h-FABP may serve as a reliable and early marker of CTx-induced cardiotoxicity. On the other hand, another study investigating the potential of h-FABP to predict 5-FU-caused cardiotoxicity failed to show similar results [45]. h-FABP is a specific [46] and early marker of myocardial injury, which should appear in the circulation within 90 minutes after myocardial damage [45,47] and should return to normal values within 24–36 hours [48]. Therefore, it was surprising to find a significantly increased h-FABP level at 7 days after 5-FU-based CTx in this study. The delayed cardiotoxicity of 5-FU-based chemotherapy might be explained by the biotransformation of 5-FU to fluoroacetate (FAC) and a-fluoro-f-hydroxypropionic acid (FHPA) in vivo [49]. FAC is a highly cardiotoxic poison [50] and FHPA is cardiotoxic at higher doses [49]. It is known, that FAC and α-fluoro-β-alanine hydrochloride (FABL), which is a precursor of FHPA, accumulate in the organism [51,52] and therefore the onset of cardiotoxicity may be delayed until the sufficient cumulative dose of cardiotoxic metabolites accumulates.

Some authors discuss that glycine may have cardiotoxic side effects itself [53,54,55]. Although, the heart seems to be a key target for glycine-induced toxicity at certain dosages that cannot be obtained when using a glycine-enriched diet [53,56,57]. Furthermore, glycine was tested in several clinical trials and none of them revealed relevant toxicity of the amino acid [56,57]. In the in vitro part of this study, the effect of glycine on cardiomyocytes’ growth and viability was evaluated and it could be shown that glycine at concentrations up to 8 mM did not have a negative effect on cardiomyocytes growth and viability. Taken together, it seems that glycine can be cardiotoxic at very high concentrations but is non-cardiotoxic and even has a cardioprotective effect against FOLFOX-induced cardiotoxicity when used in concentrations applied in the course of this experiment.

Data shown in this study state that glycine could be beneficial in cardioprotection in a CRLM FOLFOX CTx rat model. Exact pathomechanisms have to be evaluated and may comprise different modes of actions. First, glycine reduces human and rat platelet aggregation in vitro [16] and improves microcirculation [17]. Second, several recently published in vivo studies reported antioxidative features of glycine [18,19]. Since the proposed mechanisms of 5-FU-induced cardiotoxicity include vasoconstriction, endothelial injury leading to a procoagulant state, and direct myocardial cell toxicity [28,29,30,31], which was supported by the present study results, it seems that glycine may have a protective effect against all of these pathophysiologic mechanisms. Further, this study demonstrated that glycine reduces the chemotherapy-induced myocardial fibrosis, and such findings were consistent with several previous studies that showed anti-fibrotic properties of glycine in preclinical models of liver diseases [58,59] and Duchenne muscular dystrophy [60]. Since fibrosis is a key component in the pathogenesis of cardiac dysfunction and reduced ejection fraction [61], the prevention of it by glycine may play a central role in the protection of cardiac function. Moreover, besides a cardioprotective effect, glycine has other properties that are beneficial in CRLM treatment. Dietary glycine has a hepatoprotective effect against FOLFOX chemotherapy-induced liver injury [22]. Further, glycine has anti-tumorigenic properties, which were demonstrated in several experimental studies [62,63] including this experimental model as published previously [21].

Taking the results of this study together with the previously gained knowledge, it seems that glycine is a promising substance in limiting FOLFOX-induced cardiotoxicity. Further studies are needed in order to characterize the exact pathomechanisms of glycine as a chemo additive substance to FOLFOX CTx.

## 5. Conclusions

The non-toxic amino acid, glycine, showed promising cardioprotective properties in FOLFOX-based CTx in CRLM treatment. However, randomized controlled trials are needed to prove the positive effects of glycine in a human setting, bringing the promising results of this study from bench to bedside in FOLFOX-treated patients suffering from CRLM.

## Figures and Tables

**Table 1:** Genes used for quantification and primer information.

Account Number	Forward (5′->3′)	Reverse (5′->3′)	Product Length
*COL1A1 (Collagen* *1)*			
NM_053304.1	CAACCTCAAGAAGTCCCTGC	AGGTGAATCGACTGTTGCCT	77 bp
*COL2A1 (Collagen 2)*			
NM_012929.1	CAGTCGCTGGTGCTGCT	GCCCTAATTTTCGGGCATCC	76 bp
*BNP (Brain natriuretic peptide)*			
NM_031545.1	TTTCCTTAATCTGTCGCCGCT	GGATTGTTCTGGAGACTGGCT	73 bp
*ACTB (beta-Actin)*			
NM_031144.3	GCAGGAGTACGATGAGTCCG	ACGCAGCTCAGTAACAGTCC	74 bp

**Table 2:** Median white blood cell count at different time points of the study.

Group	Day 6 (×10^9^/L)	Day 14 (×10^9^/L)	Day 17 (×10^9^/L)	Day 21 (×10^9^/L)
Glycine–FOLFOX	11.1 (9.0; 12.4)	11.5 (9.5; 13.0)	4.6 (4.1; 5.2)	0.5 (0.3; 0.9)
Casein–FOLFOX	10.4 (9.6; 11.6)	11.6 (4.8; 12.7)	4.5 (3.5; 5.0)	0.4 (0.2; 0.6)
Glycine–Control	11.7 (10.6; 12.2)	11.6 (10.4; 15.8)	14.4 (12.4; 16.7)	8.4 (7.3; 9.3)
Casein–Control	11.0 (10.7; 11.9)	14.8 (10.5; 17.1)	14.9 (10.6; 17.4)	8.9 (7.3; 9.6)
Glycine–Sham	10.1 (9.7; 12.3)	9.6 (8.7; 11.2)	13.1 (12.8; 14.1)	6.8 (6.1; 7.7)
Casein–Sham	10.7 (8.0; 10.7)	10.2 (9.7; 10.4)	16.6 (13.7; 23.3)	7.0 (3.3; 8.4)

## Abbreviations

BNP	brain natriuretic peptide
CAB	chromotrope aniline blue
CBC	complete blood count
CCD	charge-coupled device
CRC	colorectal cancer
CRLM	colorectal liver metastasis
CTx	chemotherapy
ELISA	enzyme-linked immunosorbent assay
FABL	α-fluoro-β-alanine hydrochloride
FAC	fluoroacetate
FHPA	a-fluoro-f-hydroxypropionic acid
HCM	human cardiac myocyte
HGB	hemoglobin
h-FABP	heart fatty acid binding protein
LV	left ventricle
LVEDD	left ventricular end-diastolic diameter
LVEF	left ventricle ejection fraction
LVESD	left ventricular end-systolic diameter
OX	oxaliplatin
RBC	red blood cell
WBC	white blood cell
5-FU	5-fluorouracil
